# The Endocannabinoid System and Sex Steroid Hormone-Dependent Cancers

**DOI:** 10.1155/2013/259676

**Published:** 2013-11-27

**Authors:** Thangesweran Ayakannu, Anthony H. Taylor, Timothy H. Marczylo, Jonathon M. Willets, Justin C. Konje

**Affiliations:** Endocannabinoid Research Group, Reproductive Sciences Section, Department of Cancer Studies and Molecular Medicine, Robert Kilpatrick Clinical Sciences Building, University of Leicester, Leicester Royal Infirmary, P.O. Box 65, Leicester, Leicestershire LE2 7LX, UK

## Abstract

The “endocannabinoid system (ECS)” comprises the endocannabinoids, the enzymes that regulate their synthesis and degradation, the prototypical cannabinoid receptors (CB1 and CB2), some noncannabinoid receptors, and an, as yet, uncharacterised transport system. Recent evidence suggests that both cannabinoid receptors are present in sex steroid hormone-dependent cancer tissues and potentially play an important role in those malignancies. Sex steroid hormones regulate the endocannabinoid system and the endocannabinoids prevent tumour development through putative protective mechanisms that prevent cell growth and migration, suggesting an important role for endocannabinoids in the regulation of sex hormone-dependent tumours and metastasis. Here, the role of the endocannabinoid system in sex steroid hormone-dependent cancers is described and the potential for novel therapies assessed.

## 1. Introduction

Cancer is characterised by an imbalance in cell cycle regulation leading to uncontrolled cell division and reduced cell death. Previous findings, suggesting that endocannabinoids play a vital role in cell proliferation, differentiation, and/or cell survival [1, 2], indicate that modulation of endocannabinoid action may provide an effective novel therapy for the amelioration of cancer symptoms or provide a method for continuous chemoprevention against cancer. This review will focus on describing connections between the endocannabinoid system and sex steroid hormone-dependent cancers.

### 1.1. The Endocannabinoid System

Endocannabinoids and their receptors are found throughout the body: in the brain, lungs, digestive system, connective tissues, hormone releasing glands, skin/hair, bone, the immune system, and the reproductive organs. The endocannabinoid system is a multifaceted endogenous signalling arrangement that influences multiple metabolic pathways [3]. It is composed of transmembrane endocannabinoid receptors (G-protein-coupled [CB1 and CB2] receptors), their endogenous ligands (the endocannabinoids), and the proteins involved in their biosynthesis and degradation [4]. The main active ingredient of cannabis, Δ^9^-tetrahydrocannabinol (Δ^9^-THC), mediates its effects through binding and activation of CB1 [5–7] and/or CB2 receptors [8, 9]. Because THC and its analogues have been used in palliative treatments where they inhibit tumour cell growth [10], research dedicated to the potential role of THC and the modulation of the endocannabinoid system in cancer treatment has increased [10–12].

### 1.2. Endocannabinoid Synthesis and Degradation

**Figure 1:**
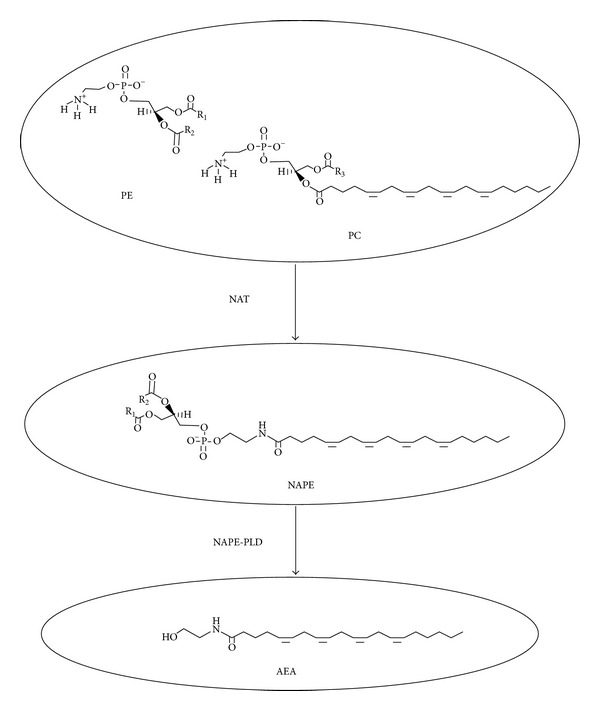
Synthesis of *N*-arachidonoylethanolamine (AEA). *N*-acyltransferase (NAT) catalyzes the transfer of arachidonic acid (AA) from phosphatidylcholine (PC) to phosphatidylethanolamine (PE) to form *N*-arachidonoyl-phosphatidylethanolamine (NAPE). NAPE is then converted into AEA in a one-step hydrolysis reaction catalyzed by the NAPE-specific phospholipase D (NAPE-PLD).

Endocannabinoids are unsaturated fatty acid derivatives, which are mainly considered to be synthesised “on demand” from phospholipid precursors residing in the plasma membrane [13] but may also be synthesised and stored in intracellular lipid droplets and released from those stores under appropriate conditions [14]. The most well-characterised endocannabinoids are anandamide (*N*-arachidonoylethanolamide, AEA) [15] and 2-arachidonoylglycerol (2-AG) [16], whose synthesis occurs through the action of a series of intracellular enzymes activated in response to a rise in intracellular calcium levels [17–19]. AEA was the first endogenous ligand identified for cannabinoid receptors and remains the most frequently investigated endocannabinoid [15]. AEA is produced via at least four separate pathways but the pathway that is most active in nonneuronal cells is the one where *N*-arachidonoyl phosphatidylethanolamine is directly converted to anandamide by the actions of *N*-arachidonoyl phosphatidylethanolamine-specific  phospholipase D (NAPE-PLD [20] (Figure 1)) that has little in common with other phospholipases [21].

**Figure 2:**
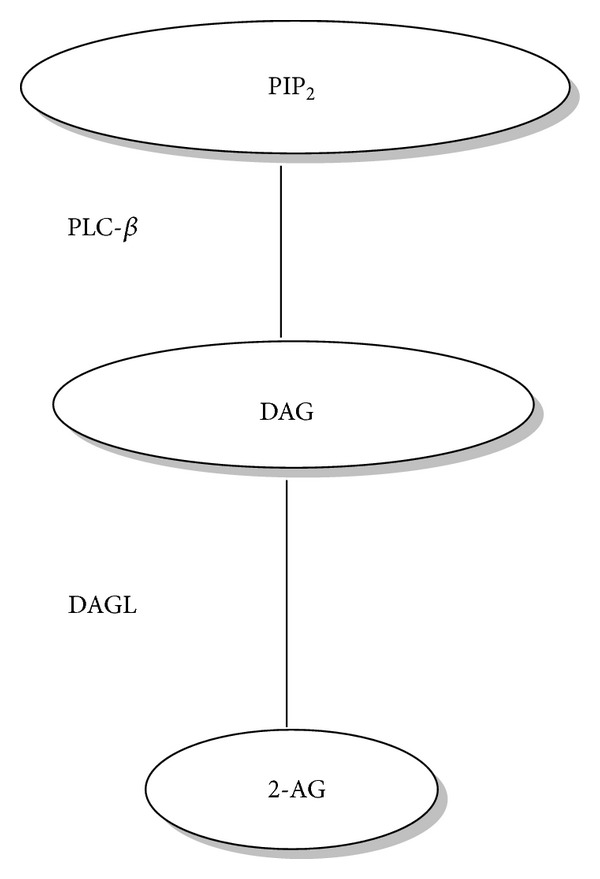
Synthesis of 2-AG. Phosphatidylinositol-4,5-bisphosphate (PIP2) is hydrolysed by phospholipase C-*β* (PLC-*β*) to form diacylglycerol (DAG). The DAG is then hydrolysed to 2-AG by diacylglycerol lipase (DAGL).

**Figure 3:**
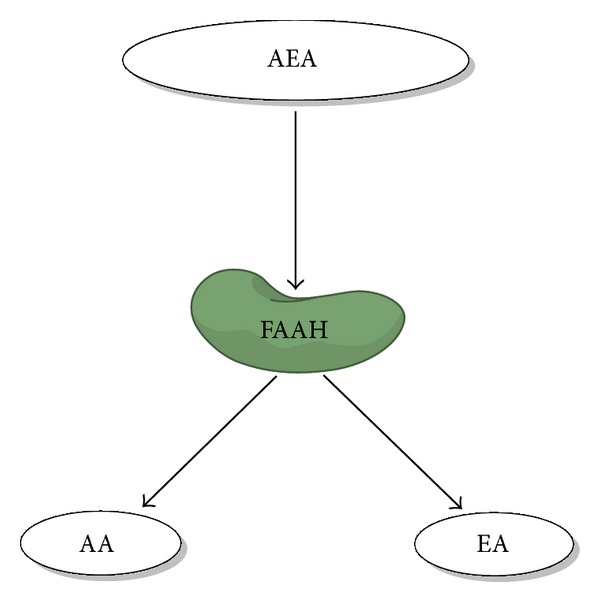
AEA is hydrolysed into arachidonic acid (AA) and ethanolamine by fatty acid amide hydrolase (FAAH).

**Figure 4:**
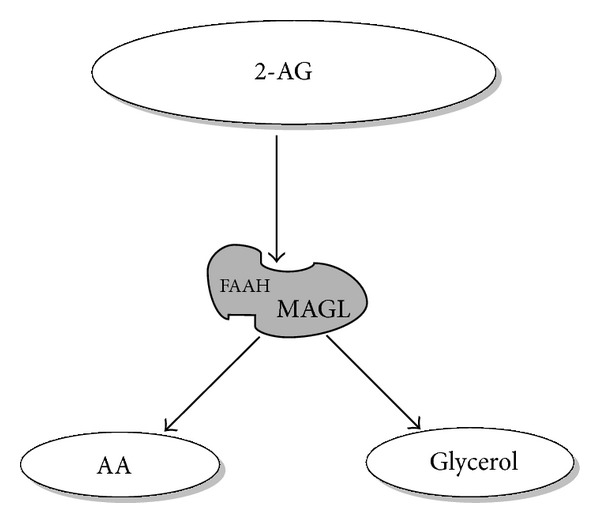
2-AG is hydrolysed by FAAH and MAGL. As indicated by the size of the letters, MAGL is the major enzyme degrading 2-AG.

The second most often studied endocannabinoid is 2-AG, which is synthesised from diacylglycerol by the sequential actions of phospholipase C and two calcium sensitive *sn*-2-selective diacylglycerol lipases (*α* and *β* DAGL) (Figure 2) [22]. When released from cells, AEA and 2-AG act in an autocrine or paracrine manner to stimulate signalling through interaction with various extracellular and intracellular receptor targets (Figure 2). To facilitate endocannabinoid reuptake and attenuate signalling, a diverse number of transport systems have been postulated, such as cellular endocytosis, simple diffusion, and specific carrier proteins [23], but none are yet proven. Both AEA and 2-AG are degraded through the action of specific enzymes; AEA is predominantly metabolised to arachidonic acid and ethanolamine by the enzyme fatty acid amide hydrolyse (FAAH-1) (Figure 3) [24] and to a lesser extent by FAAH-2 (not present in rodents). Although 2-AG is also metabolised by FAAH-1 and to a lesser extent by *α*,*β*-hydrolase-6 (ABHD6) and *α*,*β*-hydrolase-12 (ABDH12), it is not metabolised by FAAH-2; the predominant enzyme involved in its degradation is monoacylglycerol lipase (MAGL) (Figure 4) [25, 26]. Once inside the cell, AEA is considered to be moved around the cell by an intracellular FAAH-like protein (FLAT-1), that is, catalytically silent, does not bind 2-AG, and delivers it to FAAH-2 on microsomal membranes [27].

Several other endocannabinoids have been identified, including *N*-arachidonoyl-dopamine (NADA) [28], noladin ether, and virodhamine [29]. In addition, structural analogues of endocannabinoids with low affinities for cannabinoid receptors such as *N*-oleoylethanolamine (OEA), *N*-palmitoylethanolamine (PEA), *N*-stearoylethanolamine (SEA), and linoleoylglycerol have also been identified in human, rat, and mouse tissues [30, 31]. These compounds produce an “entourage effect” through being alternative substrates for FAAH and MAGL and thereby increasing the potency of endocannabinoids, such as AEA and 2-AG whose degradation by these enzymes is inhibited [32, 33]. Endocannabinoids may also undergo oxidative metabolism by a number of fatty acid oxygenases, such as cytochrome P450 enzymes (CYP450) [34, 35], lipoxygenases (LOX) [36, 37], and cyclooxygenase-2 (COX-2) [38, 39]. Stimulation of CYP450s, LOXs, and COX-2 in tumour cells and inflammation sites could thus reduce the levels of naturally occurring antiproliferative and anti-inflammatory mediators [40, 41].

### 1.3. Receptors

Two subtypes of cannabinoid receptors belonging to the Gi/o family of seven trans-membrane G-protein-coupled receptors (GPCR) have been described. The first, CB1, or the central receptor was first described as being predominantly expressed in the central nervous system but is also present in a variety of peripheral tissues at much lower levels [42–44]. The second, CB2, or the spleen-type receptor was originally isolated from splenic cells and is primarily expressed in immune and blood cells, although it has also been found in various brain areas [45] and other tissues [46, 47]. Both receptors are distributed in human tissues including the brain, testis, sperm, leucocytes, placenta, fetal membranes, endothelial cells, anterior eye, pituitary gland, breast, and reproductive tissues [17, 48–54]. Surprisingly, they share little sequence homology, only 44% at the protein level or 68% in the trans-membrane domains, which are thought to contain the binding sites for cannabinoids [55]. Pharmacological studies have strongly suggested the existence of novel cannabinoid receptor subtypes [56, 57], and recently two orphan G-protein-coupled receptors (GPR55 and GPR119) have been proposed as cannabinoid receptors. GPR55 has been identified in the brain and peripheral tissues such as the gut, spleen, and adrenals, and, of the endocannabinoids, 2-arachidonoylglycerol (2-AG) and PEA have the greatest affinity for this receptor. GPR119 on the other hand has a narrow distribution having been described predominantly in the pancreas and intestinal tissues. The endocannabinoid with greatest affinity for GPR119 is OEA [58]. Cannabinoids can also inhibit various types of calcium channels [59, 60] and activate certain potassium channels [61]. In addition, the transient receptor potential vanilloid subtype 1 (TRPV1), a ligand-gated Ca^2+^ permeable ion channel, usually activated by stimuli such as acidity and heat and involved in the transmission and modulation of pain [62], is also activated by both AEA and acyl dopamine referred to as endovanilloids in this context [62]. Lately, the peroxisome proliferator-activated receptors (PPARs) have been included in the lists of the endocannabinoid targets, as they are stimulated by endocannabinoids under both physiological and pathological conditions [63].

## 2. Endocannabinoids and Cancer

Adjuvant cannabinoid use in the treatment of adverse side effects from chemotherapy, such as neuropathic pain, loss of appetite, nausea, and vomiting, is the most studied potential therapeutic application for these compounds [64]. Beyond the palliative effects induced by cannabinoids, these molecules and endocannabinoids are increasingly recognised for their role in the regulation of the key processes involved in the development of cancer. For example, the endocannabinoid system is reported to induce apoptosis [11, 65, 66], cell cycle arrest [67–69], and the inhibition of angiogenesis and metastasis [70–72] in animal models and cell lines. Further research is however needed to substantiate these antineoplastic effects in humans. Furthermore, there is a suggestion that endocannabinoid signalling in the tumorigenic cell differs from that of its “normal” counterpart.

### 2.1. The Endocannabinoid System: Cancer versus Normal Cell

When considering the development of a novel anticancer treatment that “selectively targets tumour cells,” thereby improving the therapeutic index of anticancer strategies, a comparison of the action of the drug in cancer cells with respect to that in normal cells represents a crucial step that must be carefully explored. Where this shows significant effects on the cancer, but not on the normal cells, such a drug will have potential benefits.

Evidence suggests that the actions of the endocannabinoid system indeed are selective in cancerous rather than in noncancerous cells [73–75]. These are affected by different components of the endocannabinoid system and result in a wide range of actions. In cancer cells such as those of the breast, melanoma, lymphoma, pancreas, and thyroid, there is increased sensitivity to endocannabinoids due to an increased level of endocannabinoid receptors in these cells compared to normal cells found in adjacent tissue obtained from the same specimen [73, 75–81]. For example, met-fluoro-anandamide (Met-F-AEA) increases the levels of CB1 receptors in both K-ras-transformed FRTL-5 (KiMo1) cells and in KiMo1-derived tumours in nude mice, whereas in FRTL-5 cells (a thyroid-differentiated epithelial cell line), Met-F-AEA produced downregulation of CB1 receptors [75]. Treatment of human prostate cancer (LNCaP) cells with the CB agonist WIN-55,212-2 showed significantly higher expression of both CB1 and CB2 receptors in these cells when compared to normal cells and interestingly a significant decrease in cell viability when treated with 1–10 *μ*M of WIN-55,212-2 for 24–48 hours, whilst similar doses had no effect on prostate epithelial (PrEC) cells [78].

Furthermore, the RAS-MAPK/ERK pathway in brain cells is one signalling pathway, which has been reported to be differentially regulated by cannabinoids in the cancerous cell when compared to the normal cell [82], where THC induces ceramide synthesis and glioma cell death via a CB1-mediated effect, whilst astrocytes are protected from ceramide-induced sensitisation to oxidative stress-related damage [83]. Similarly, proapoptotic and antiproliferative effects of cannabinoids on cancer cells and not on healthy tissue have been recorded in animal studies [65, 76], whilst cultured oligodendroglial cells are protected from various proapoptotic stimuli [84]. Furthermore, THC induces apoptosis in several human cancer cell lines [11, 74, 85], whilst endocannabimimetic substances inhibit the proliferation of KiMol cells more robustly than FRTL-5 cells and *in vivo*, Met-F-AEA inhibits the growth of KiMol-induced tumours in athymic mice, an effect that was accompanied by a reduction in p21ras action [75]. In addition, ligand-induced activation of CB2 receptors reduces human breast cancer cell proliferation, whereas in normal breast tissue the expression of CB2 receptor was significantly less and the proliferation was thus much less affected [78].

Furthermore, elevated levels of AEA and 2-AG have been documented in several other cancerous tissues when compared to normal healthy counterparts, such as prostate and colon cancer, endometrial sarcoma, pituitary adenoma, and highly aggressive human cancer tissues [52, 53, 86, 87]. Recently, an increase in FAAH expression in prostate cancer cell has been reported when compared to that in the noncancerous prostate cell [88]. The expression level of MAGL is higher in androgen-independent versus androgen-dependent human prostate cancer cell lines and RNA-interference disruption of MAGL impairs prostate cancer aggressiveness [89], suggesting that 2-AG has a role to play in the aggressiveness of some types of prostate cancer.

The effect of endocannabinoids on tumorigenesis may depend on the stage of differentiation of the malignant tissues under investigation. In the human colon cancer cell line Caco2, endocannabinoids failed to show any proliferative effect via CB1 receptors in differentiated cells [90]; however, in undifferentiated cells, cannabinoids were strongly antiproliferative via CB1 and this was not because of alterations in the levels of CB1 receptors. Intriguingly, an alteration in CB1 glycosylation that probably affected cell signalling was suggested [90]. However, similar elevations in CB receptor glycosylation could not be found in human bladder, pancreas, or small intestine cancer cells [86, 91], suggesting this is not the main reason for the antiproliferative effects observed in the undifferentiated Caco2 cells. The small difference in CB receptor expression, although not significant in these studies, could possibly be applicable in other hormone-dependent cancer cells.

### 2.2. Endocannabinoid Receptor Signalling Actions and Consequences in Cancer

The variability of endocannabinoid effects in different tumour models is highly incongruous and may be a consequence of the differential expression of cannabinoid receptors, where it is envisioned that differential expression of cannabinoid receptors between cancerous and normal tissues may play a determining role in the progression and/or inhibition of malignancy. For example, high levels of CB1 and CB2 mRNA were detected by *in situ* hybridization in well-differentiated human hepatocellular carcinoma and in cirrhotic liver samples, while the expression of these receptors in poorly differentiated hepatocellular carcinoma was low [92]. In addition, increased expression of CB1 and/or CB2 has been noted in human mantle cell lymphoma [80, 81], breast cancer [79], acute myeloid leukaemia [93], hepatocellular carcinoma, and prostate cancer cell lines; however, the levels of both receptors were similar in malignant and nonmalignant human astroglial cancer cells [94] and in malignant and nonmalignant nonmelanoma skin cancer cells [70].

In general, a relationship between CB receptor expression and the outcome of cancer has been documented. In astrocytoma cells, for example, it has been shown that 70% of cells express CB1 and/or CB2 with the extent of CB2 receptor expression correlating directly with the degree of tumour malignancy [66], whilst in gliomas a higher expression of CB2 receptor compared to CB1 receptor was found and related to tumour grade [66]. In addition, tumour-associated endothelial cells demonstrated immunoreactivity for CB1 receptors similar to that of the cancer cells [95]. Similarly, increased expression of both CB1 and CB2 receptors has been documented in non-Hodgkin lymphoma when compared to reactive lymph nodes [80], whilst CB1 expression is increased in mantle cell lymphoma [96]. In contrast, a significantly reduced expression of CB1, but not of CB2, was noted in colon cancer compared with the normal adjacent mucosa [97]. Taken together, these studies imply a role for CB1 and CB2 receptors and their expression in relation to disease prognosis and outcome and that this is greatly dependent on the type/specific cancer being studied.

In breast carcinoma, a relationship between CB2 expression, the histological grade of the cancer, and other markers of prognostic and predictive value, such as ErbB2/HER-2 oncogene, oestrogen, and progesterone receptors, has been reported [98]. CB1 receptor expression in the human prostate cancer cell lines LNCaP (androgen-sensitive), DU145 and PC3 (androgen-insensitive) has been reported to be higher than in the normal human prostate epithelial cells [78]. This was confirmed in prostate cancer tissues where the expressions of CB1 and TRPV1 receptors were upregulated and furthermore correlated with increasing cancer grade [99]. Moreover, the level of CB1 receptor expression in cancer specimens has been shown to correlate with the disease severity at diagnosis and outcome [100]. In human pancreatic cancer, higher levels of CB1 receptor expression are related to a shorter survival time (median 6 months) than lower CB1 receptor (median 16 months) [91]. In contrast, the overexpression of CB1 and CB2 receptors found in human hepatocellular carcinoma was associated with improved prognosis [92].

The mechanism by which endocannabinoid receptor expression is modulated in relation to cancer has not been fully examined; however, several studies have revealed important evidence for further relationships between cannabinoid receptors and cancer, where transcription factor involvement has been postulated. Indeed, it has been shown that THC induces a CB2-receptor-dependent transcription of the CB1 gene in human T cells and T cell lymphoma lines, mediated via IL-4 release through activation of the transcription factor STAT6 [101]. In addition, expression of CB2 is induced following the oral administration of specific *Lactobacillus* strains in colonic epithelial cells, through the NF-*κ*B pathway [102], whilst it has been reported that CB1 receptor expression in human colon cancer was induced by 17*β*-oestradiol through an oestrogen-receptor-dependent mechanism [103]. In alveolar rhabdomyosarcoma, CB1 receptor expression has greatly increased and this was evident in chromatin immunoprecipitation studies, which have demonstrated that the CB1 gene is a transcriptional target of PAX3/FKHR, a chimeric transcription factor found in this condition [104]. Another hypothesis, that has been examined, is that alternatively spliced isoforms of CB1 (CB1a and CB1b), which could reflect differences in its functionality in normal and cancerous tissues, are responsible for the variability in the response described above [105].

### 2.3. The Endocannabinoid System and Sex Steroid Hormones

**Figure 5:**
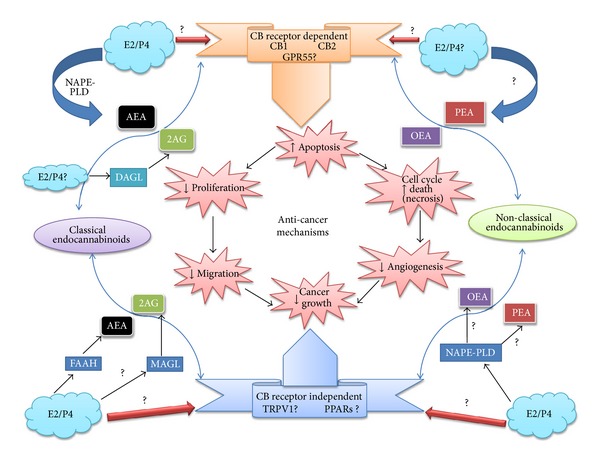
Known and putative interactions between gonadal sex steroids and the endocannabinoid system in sex hormone-dependent cancers. Endocannabinoid synthesis occurs through the actions of specific enzymes (blue boxes and broad arrows) through enzymatic hydrolysis of membrane specific NArPE lipid precursors. The role of the sex steroid hormones oestradiol (E2) and progesterone (P4) (blue clouds) is only speculative. The classical (anandamide (AEA) and 2-arachidonoylglycerol (2-AG)) and nonclassical endocannabinoids (*N*-oleoylethanolamine (OEA) and *N*-palmitoylethanolamine (PEA)) independently or collectively bind at both classical (CB1, CB2, and GPR55) and nonclassical (TRPV1 and PPAR) receptors (ribbons) to affect anticancer mechanisms (pink starbursts in the centre of the figure), which may be linked (arrows) or may not be linked to each other, but all result in reduced cancer cell mass. Known interactions between sex steroid hormones and the endocannabinoid system in the anticancer mechanisms are shown, whereas speculative or unknown interactions are indicated by the presence of a question mark. Up and down arrows indicate either an increase or decrease in the activity of that particular anticancer mechanism.

The endocannabinoid system is widespread throughout the central nervous system (CNS) and peripheral regions and regulates a large range of physiological functions and behaviour. The same can also be said of the sex steroid hormones. As stated above, there is evidence suggesting that the two systems interact extensively (Figure 5).

#### 2.3.1. The Role of Progesterone

Progesterone is a C-21 steroid hormone that is produced predominantly by the ovarian corpus luteum after ovulation and is possibly involved in the regulation of endocannabinoid signalling. Progesterone has been shown to upregulate human lymphocyte FAAH activity through the transcription factor Ikaros [106, 107] and thereby decreases plasma AEA levels [108]. Whether this is a general phenomenon or a T cell specific effect needs clarification since the expression of Ikaros transcription factors seems to be confined to the T cell [109]. Furthermore, while progesterone increases FAAH expression and its activity in immortalized human lymphoma U937 cells, but not in immortalized human neuroblastoma CPH100 cells [110], it has been reported to have a minimal effect on EMT, NAPE-PLD, and CB1 expression in lymphocytes [106, 107]. Progesterone has also been documented to downregulate uterine NAPE-PLD expression in mice, leading to a decrease in tissue AEA levels [111]. In the pregnant mouse uterus, it has also been reported to downregulate FAAH activity [112], and when taken together with reduced NAPE-PLD expression in mice these data suggest that the NAPE-PLD  : FAAH activity ratio in the mouse uterus may be key to the regulation of local AEA levels and thus maintenance of pregnancy or endometrial pathologies, such as cancer. At the same time, the actions of progesterone on local AEA levels in the rat uterus are more complex with progesterone stimulating its production in the ovariectomised animal [113]. These authors concluded that “…the effect of ovarian hormones on the synthesis of anandamide depends on different physiological conditions, (including the) oestrous cycle and early pregnancy, and on the presence of the activated blastocyst…,” but the precise feedback mechanisms that might be involved are unknown. For example, treatment with either CB1 or CB2 receptor agonists reduces the levels of serum progesterone, corpus luteum weights, corpus luteum LH receptor mRNA content, and corpus luteum LH receptor density in sheep [114]. It has also been reported that the levels of serum progesterone and LH content are decreased following chronic administration of AEA in pregnant rats [115], suggesting that both positive and negative feedback loops are involved in the coregulation of endocannabinoid and progesterone function.

#### 2.3.2. The Role of Oestrogen

Oestrogens are also steroid hormones, produced predominantly by the ovarian follicle during the early stage of the menstrual and oestrous cycles. Once inside the cell, oestrogens bind to and activate specific oestrogen receptors, resulting in the regulation of the expression of multiple gene targets involved in cellular proliferation, apoptosis, and autophagy [116]. Although FSH and LH stimulate the synthesis of oestrogen in the ovaries, there are other nonovarian sources of oestrogens such as the breast, the adrenal glands, and the liver, but the levels produced are relatively small and probably only have local actions [117]. The most potent growth stimulating oestrogen is 17*β*-oestradiol (E2), which has been linked directly and indirectly with the endocannabinoid system, where E2 stimulates NAPE-PLD and inhibits FAAH synthesis and directly stimulates the release of AEA from endothelial cells [108, 118, 119]. By contrast, another study revealed that NAPE-PLD is downregulated in the uterus by oestradiol, suggesting that it results in decreased anandamide levels, although this was not directly tested [111]. However, other studies revealed that E2 decreases the activity of FAAH in the mouse uterus [108, 118], whilst also regulating the expression of FAAH [112]. Moreover, in cancers, such as glioma, breast, and colon, oestrogens appear to regulate the ECS [120]. Evidence of cannabinoid and oestrogen receptor coexpression has been documented in colorectal carcinoma and normal colonic epithelium [90] and in the human anterior pituitary gland [121], where E2 regulates CB1 mRNA expression, a feature it also shared within the rat hypothalamus [121]. These differential effects of oestrogens on components of endocannabinoid signalling pathways reveal an intricate interaction which may play an important role in sex steroid hormone-dependent tumours.

## 3. The ECS and Breast Cancer

Breast cancer is the most common cancer in women [122]. It is dependent on hormones, such as oestrogen and progesterone, for initial growth and survival. Risk factors for the development of breast cancer include lower fertility, nonbreastfeeding, genetic predisposition, higher hormone levels, and iodine deficiency [123]. Bones, lungs, and lymph node are among the sites where breast cancer cell may spread [124, 125], but the cancer itself normally develops from breast tissues surrounding the milk ducts (ductal neoplasm) or breast lobules (lobular neoplasm) [126]. Cannabinoid receptors have been documented to be present in breast tissue; CB1 immunoreactivity was expressed in 28% of human breast cancer samples [127] and immunohistochemistry studies have revealed the presence of CB1 in 14% of human breast cancer tissues expressing ErbB2, which is a member of the epidermal growth factor (EGF), but interestingly with no relationship between CB1 and ErbB2 expression [128]. CB1 receptors have also been documented in human breast tissues using Western blot, immunofluorescence, and/or RT-PCR techniques as well as in various breast cancer cell lines (T-47D, MCF-7, MDA-MB-231, TSA-E1, and MDA-MB-468) [69, 103, 127, 129, 130].

In contrast, CB2 immunoreactivity was documented in 72% of human breast tissues [128], and is present in 91% of ErbB2-positive cancer tissues (in contrast to CB1 receptor), suggesting a relationship between CB1 receptor and the ErbB2-positive cancer cell phenotype [128]. CB2 immunoreactivity was noted in 35% of human breast cancer tissues [127] while CB2 receptors were expressed in a variety of breast cancer cell lines (T-47D, MCF-7, MDA-MB-231, MDA-MD-468, EVSA-T, and SkBr3) and human breast tissues as determined by Western blot, RT-PCR, and/or immunofluorescence techniques [69, 103, 127, 129, 130]. In addition, FAAH transcripts are present in the EFM-19 and MCF-7 cancer cell lines, as determined by northern blotting [131] and RT-PCR [132] techniques, whilst GPR55 is highly expressed in the MDA-MB-231 and MCF-7 breast cancer cell lines [133].

Recent research has identified a role for the endocannabinoid system in the regulation of breast cancer growth, with induction of apoptosis and control of cancer neovascularization in breast cancer being key control points [127, 128, 130, 134] and importantly a critical relationship with oestrogen [116]. These effects are achieved through a variety of mechanisms; for example, CBD seems to involve direct TRPV1 activation and/or CB2 indirect activation (via FAAH), induction of oxidative stress [130], and the ability to decrease ID-1, an inhibitor of basic helix-loop-helix transcription factor. The expression of ID-1 in breast cancer cells was associated with its efficacy in reducing proliferation, migration, and invasion [135]. In these types of cell lines, the endocannabinoid system induces growth arrest by downregulating prolactin receptor expression [67]. Therefore, breast cancer proliferation depends on signalling via the CB1 receptor, which has been revealed to downregulate the prolactin receptor and indirectly inhibit cell growth [67]. Furthermore, these effects were also noted when FAAH activity was blocked [67], suggesting that AEA is somehow involved in this process. In addition, the antiproliferative effects of the endocannabinoid system were shown to be mediated through downregulation of the high affinity NGF receptor [69]. Similarly, the ECS inhibits breast cancer growth *in vivo*, by acting through the CB1 receptor [134]. Finally, a recent study showed that CBD induces a concentration-dependent cell death of both oestrogen receptor-positive and oestrogen receptor-negative breast cancer cells through a mechanism involving a CB1-, CB2-, and TRPV1-independent receptor activation [116]. Furthermore, the gender-specific actions of E2 in the hippocampus, where the ER*α*-specific inhibition of CB1-dependent signalling in a subset of neurons occurs only in female and not in male mice, whilst ER*β*-specific stimulation occurs in both genders, suggest that there may be a complex interaction between oestrogen and cannabinoid signalling, at least in the rat hippocampus [136]. Considering that the E2-activated ER*α* promotes human breast cancer cell growth [137] and the E2-activated ER*β* receptor inhibits human endometrial cancer cell growth [138] and that cannabinoids may affect both breast and endometrial cancer growth and development, then a potential interaction between these two signalling pathways seems plausible and should be investigated further.

## 4. The ECS and Prostate Cancer

Prostate carcinoma is the second most common cancer diagnosed in men [122]. Factors such as diet, genetic predisposition, medical exposure to hormones, and viral infections are all implicated in the incidence of prostate cancer [139]. The majority of prostate cancers have a slow progression, although cases of aggressive prostate cancer do occur. Common sites of prostate metastasis are particularly in the bones and lymph nodes [139, 140]. CB1 expression is upregulated in prostate cancer tissues [99] and the levels of the receptor are associated with cancer severity and outcome [100]. In addition, RT-PCR, Western blot, and immunofluorescence studies have shown that prostate cancer cell lines, PC-3, DU-145, LNCaP, CWR22Rv1, and CA-HPV-10, and human prostate tumour tissues express CB1 [69, 78, 87, 99, 100, 141, 142]. CB2 receptors are also present in the same prostate cancer cell lines [69, 78, 87, 142] and these cancer cell lines express higher levels of CB1 and CB2 than benign prostate epithelium [78]. In addition, FAAH expression has been demonstrated in the prostate cancer cell lines (PC-3, DU-145, and LNCaP) and human prostate cancer tissues [88, 97, 125, 142, 143]. GPR55 is also expressed in the PC-3 and DU-145 prostate cancer cell lines [144]. Thus, various cannabinoid receptor subtypes and endocannabinoid hydrolysing enzymes are known to be located in prostate tissue and synthetic cannabinoids, endocannabinoids, and related compounds appear to inhibit prostate tumour cell proliferation and induce apoptosis via CB1 and/or CB2 receptor activation.

Prolactin (PRL) is necessary for the prostate to be completely formed. Prolactin is a hormone produced by the anterior pituitary gland in lactotroph cells and its gene is located on chromosome 6 [145]. Prolactin plays a vital role in prostate cell proliferation, differentiation, and survival, in normal as well as in malignant cells [146]. This has led to the suggestion that prostate cancer may express prolactin receptors and proliferate in response to prolactin levels and this response can be inhibited by cannabinoids. When induced by exogenous PRL, the proliferation of prostate DU-145 cells was potently inhibited (IC50 = 100–300 nM) by anandamide, 2-AG, and HU-210. Anandamide was also noted to downregulate the levels of prolactin in DU-145 cells [69].

Several intraepithelial or invasive prostate cancers have exhibited increased expression of epidermal growth factor receptor (EGF-R), tyrosine kinase, and EGF. The EGFR levels can be downregulated by micromolar concentrations of AEA via the CB1 receptor and this results in the inhibition of proliferation at day 3 and cell death by apoptosis/necrosis on day 5, with this effect being manifest through both CB1 and CB2 receptors [147]. Cannabinoids have also been documented to downregulate androgen receptor expression and prostate specific antigen (PSA) [148]; however, the sensitivity to cannabinoids seems to be inconsistent in different prostate cancer cell lines, even in the presence of CB receptors [128, 147, 148].

## 5. The ECS and Endometrial Cancer

Worldwide, endometrial cancer is the seventh most commonly diagnosed malignancy [149] and the 4th most common gynaecological cancer diagnosed in 2008 in the UK [150]. Endometrial cancer refers to several types of malignancies that arise from the endometrium, and early menarche, late menopause, obesity, nulliparity, and the use of oestrogen-only hormone replacement therapy have all been identified as risk factors for the development of endometrial carcinoma, which suggest that greater lifetime exposure to oestrogen, unopposed by progesterone, plays a vital role in the aetiology of endometrial cancer [151, 152]. Exposure to endogenous or exogenous oestrogens and the use of unopposed progesterone lead to an increase in the mitotic activity of endometrial epithelial cells and increased DNA replication and repair errors, which in turn leads to various somatic mutations that may ultimately result in endometrial hyperplasia, which may finally result in the development of malignancy [153, 154]. Chronic inflammation has also been implicated as a vital player in the relationship between obesity, menstrual disorders, and endometrial cancer [153]. Moreover, conditions such as uterine fibroids and endometriosis may produce an increased risk of endometrial cancer because these disorders have been linked to both pelvic inflammation [155–157] and an excess of oestrogen [158, 159]. Based on clinicopathological and molecular characteristics, there are two types of endometrial cancers. The first is the type I or oestrogen-dependent endometrioid carcinomas (EECs), which constitute approximately 80% of the cases of endometrial carcinoma. These tumours express oestrogen (ER) and progesterone (PR) receptors and arise in younger pre- and postmenopausal women [160]. Type I is also strongly associated with either endogenous or exogenous unopposed oestrogen exposure and is usually of low grade and characterised by a favourable prognosis. The second group, the type II or nonendometrioid endometrial carcinomas (NEECs), are comprised of the high grade papillary serous and clear cell carcinomas [161]. These arise in relatively older women and are not usually preceded by an unopposed oestrogen exposure history but have an aggressive clinical course and a worse prognosis than type I cancers [161].

The expression of endocannabinoid receptors in different endometrial cancer tissues/cell lines has been described, where CB2 expression was detected by immunohistochemistry only in the endometrial cancer cells and not in the normal endometrial tissue taken from the same biopsy [162]. Immunoblotting analysis showed that CB2 protein expression was significantly elevated in the endometrial cancer tissues when compared to healthy endometrial tissues [162] and no significant differences were noted in CB1 expression [162]. A mass spectrometry study showed selective upregulation of 2-AG in endometrial cancer tissues compared to healthy endometrial tissues, whilst no significant increases in the levels of AEA or PEA were noted [162]. Similarly, immunoblotting revealed a selective downregulation of MAGL expression in endometrial cancer tissues compared with healthy tissue and, interestingly, there were no significant differences in FAAH protein expression [162]. Furthermore, evidence suggests that CB2 receptor regulation is dysregulated in endometrial cancer, because CB2 levels were significantly higher in the AN3CA human endometrial carcinoma cell line compared to control cells when transfected with a plasmid containing the cDNA for the endocannabinoid receptor CB2 [162]. From these data, it has been concluded that CB2 receptors might play a vital role in the growth of endometrial cancer [163].

Recent research has shown that the complete endogenous pathway for CB2 was altered significantly in endometrial adenocarcinoma, which may thus be one of the underlying factors for endocannabinoid system regulation in the aetiology of endometrial cancer. The marked elevation in CB2 receptor expression and 2-AG in endometrial cancer tissues might be due to the underlying imbalance in the oestrogen/progesterone ratio, which is one of the aetiological factors for the development of endometrial cancer [162], but this has not been fully tested. What has been tested was the effect of CB2 elevation in transfected AN3CA cells where CB2 caused a 40% reduction of cell mitochondrial function when compared to the control cells [163]. This effect was not improved by the CB2 receptor agonist, JWH133, but was fully prevented by the CB2 receptor antagonist SR144528.

The specific increased expression of CB2 receptors in only the tumour developing cells might represent a novel indicator for the diagnosis of endometrial cancer. Therefore, selective CB2 agonists might represent the foundation for the development of new antitumour compounds against endometrial carcinoma, because they have the ability to kill the affected cancer cells without damaging their normal counterparts [164].

## 6. Conclusions and Future Directions

Currently available studies suggest that the endocannabinoid system may be targeted to restrain the development and progression of breast, prostate, and endometrial carcinoma. The endocannabinoid system exerts a variety of interesting effects that are dependent on the cell line and/or tumour type under investigation, where the ECS, for example, inhibits cancer cell proliferation, angiogenesis, cancer growth, metastasis, and apoptosis. The prevailing data suggest that an imbalance in the endocannabinoid system and its interaction with sex steroid hormone homeostasis may promote cancer development, proliferation, and migration. Therefore, for this vital reason, and although it is early days, the endocannabinoid system has become an attractive novel target for pharmacological intervention in the fight against many hormone-related cancers.
